# Patient Experiences of Access, Stigma, and Trust and Preferences for Non-Opioid Drug Delivery Systems in Chronic Pain Management

**DOI:** 10.3390/healthcare14203379

**Published:** 2026-10-10

**Authors:** Rashed M. Almuqbil, Mostafa Shaban, Sayed Ibrahim Ali

**Affiliations:** 1 Department of Pharmaceutical Sciences, College of Clinical Pharmacy, King Faisal University, Al-Ahsa 36362, Saudi Arabia; ralmuqbil@kfu.edu.sa; 2 Gerontological Nursing Department, Faculty of Nursing, Cairo University, Cairo 11562, Egypt; 3 Department of Family and Community Medicine, College of Medicine, King Faisal University, Al-Ahsa 31982, Saudi Arabia

**Keywords:** chronic pain, opioid analgesics, health policy, opioid regulation, stigma, trust, non-opioid analgesia, topical and transdermal drug delivery, acceptability, qualitative research, Saudi Arabia

## Abstract

**Highlights:**

**What are the main findings?**

- Participants described restrictive opioid access in terms of logistical burden, stigma, concealment and erosion of institutional trust, which the research team interpreted as interconnected experiences.
- Patients expressed anticipated, hypothetical acceptance of topical and transdermal non-opioid options, conditional on affordability, reliable supply, credible efficacy, devotional fit and endorsement by a trusted clinician.

**What are the implications of the main findings?**

- Opioid stewardship in low-consumption settings may benefit from balancing safeguards against excess with safeguards against undertreatment where opioids are indicated and from dispensing processes that avoid public marking; these proposals require evaluation.
- A defined nurse-led continuity role is proposed as a potential strategy for strengthening explanation and follow-up; its effects on disclosure, uptake of alternatives and pain outcomes require prospective evaluation.

**Abstract:**

**Background/Objectives:** Chronic pain is common in Saudi Arabia, where opioid analgesics are prescribed and dispensed within a tightly regulated, electronically monitored controlled-substance framework. Qualitative evidence on how patients experience restrictive access comes almost entirely from high-consumption countries; to our knowledge, no study has examined these experiences among Saudi adults with chronic non-cancer pain or linked them to how patients appraise non-opioid alternatives. This study explored opioid access, stigma and therapeutic trust among adults with chronic non-cancer pain in Al-Ahsa and examined the anticipated acceptability of non-opioid drug-delivery systems. **Methods:** A qualitative interview study was conducted across seven public and private health settings in Al-Ahsa (May–June 2026). Thirty adults with chronic non-cancer pain were purposively sampled for maximum variation; 12 clinicians were also interviewed to contextualise the service conditions patients described, with patient accounts remaining the analytic focus. Sample adequacy was judged using information power, i.e., the capacity of a specific sample and focused dialogue to address a narrow aim. Data were analysed using reflexive thematic analysis and reported according to COREQ. **Results:** Four themes were constructed: navigating a restricted access pathway (“A door with many locks”); enacted and anticipated stigma (“As if I came to steal”); institutional distrust alongside trust in individual clinicians (“The person, not the system”); and conditional, anticipated acceptability of topical and transdermal non-opioid formats (“Something I can put on my skin”). **Conclusions:** Participants described restrictive opioid pathways in terms of access burden, stigma and disrupted trust. Their stated openness to non-opioid formats was hypothetical and conditional on affordability, reliable supply, credible efficacy, devotional compatibility and endorsement by a trusted clinician. Less stigmatising dispensing, explained dose reduction and a nurse-led continuity role are proposed as strategies requiring prospective evaluation.

## 1. Introduction

Chronic pain is among the most common reasons for seeking health care and one of the largest contributors to years lived with disability worldwide [1,2,3]. In Saudi Arabia, a national survey of 24,265 adults reported a chronic pain prevalence of 46.4%, with roughly half of respondents dissatisfied with available pain services [4]; a meta-analysis of 124 Saudi studies estimated a one-year low back pain prevalence of 52.3% [5]; and clinic-based data show a female predominance among patients presenting with pain [6]. Chronic pain is therefore a mainstream population health problem in the Kingdom.

How chronic pain is treated depends heavily on the regulatory architecture surrounding analgesics, but two clinical contexts need to be distinguished. For cancer and palliative pain, where opioids are an established treatment, the defining global problem is deficit rather than excess: most of the world’s population has little access to opioid analgesia for serious health-related suffering [7,8], policy responses designed for high-consumption settings risk deepening this “global pain divide” [9,10], and regulatory burden, knowledge deficits and supply constraints have been identified as the dominant health-worker-level barriers to opioid access for cancer pain [11]. Across 19 Arab countries, morphine consumption represents under 1% of world consumption, and most countries restrict prescribing to licensed specialists, mandate special prescription forms and prohibit refills [12,13]. Long-term opioid therapy for chronic non-cancer pain raises a different question. Systematic reviews have found insufficient evidence on long-term effectiveness, small average benefits for pain and function in shorter-term trials, and well-documented dose-related harms [14,15], and a 12-month randomised trial in patients with chronic back pain or hip or knee osteoarthritis pain found opioid therapy not superior to non-opioid medication therapy for pain-related function [16]. Current guidance accordingly recommends maximising non-opioid therapies and initiating or continuing opioids only when expected benefits outweigh risks [17]. In this population, clinicians’ reluctance to initiate or maintain long-term opioid therapy may reflect legitimate clinical uncertainty rather than irrational fear. The present study therefore does not ask whether patients with chronic non-cancer pain should receive more opioids but how restriction, whatever its clinical justification, is organised, communicated and experienced.

Saudi Arabia regulates narcotic and psychotropic medicines within a long-standing national control framework, and oversight has intensified in recent years. The reclassification of pregabalin and gabapentin as controlled substances under national electronic monitoring curbed over-prescribing but, according to an expert review, was followed by physician hesitancy, treatment delays and stigma for patients with legitimate neuropathic pain [13]; physicians have described comparable effects of control arrangements for codeine [18]. In February 2025, the Ministry of Health announced a national platform for the electronic prescribing, dispensing and monitoring of narcotic, psychotropic and controlled medicines across health facilities, replacing paper prescriptions [19]. Under this platform, prescribing is limited to consultant and specialist-grade physicians acting within their specialty, and controlled-medicine prescribing and dispensing over the preceding 90 days are visible across facilities; at the supply-chain level, the Saudi Food and Drug Authority operates a separate electronic Controlled Drugs System for authorised organisations handling these products [20]. The national architecture during the study period was therefore electronic, specialist-based and centrally monitored. Evidence on the patient-level consequences of restriction, however, comes overwhelmingly from North America, where tapering and discontinuation of long-term opioid therapy have been associated with overdose and mental health crisis [21,22,23], although ecological analyses show that the population-level picture is contested [24]. Restriction is nonetheless experienced by individual patients, and that experience has rarely been examined outside high-consumption countries.

Qualitative and survey research from these countries converges on three observations. First, patients with chronic pain report being labelled as drug-seeking, particularly in emergency departments, and respond through concealment and self-advocacy [25,26]; stigma operates at patient, clinician and community levels [27], perceived by patients and physicians alike [28] and, in Nigeria, has been shown to operate through disciplinary prescribing and dispensing practices [29]. Second, trust and communication are associated with outcomes: shared decision-making is associated with less prescription opioid misuse, partly through trust in the provider [30]; patients’ experience of opioid management turns on expectation and communication [31]; and tapers are experienced as harmful when inadequately explained, with the therapeutic relationship shaping how they are understood [32,33,34]. Third, the credibility of pain testimony is unevenly distributed, being discounted through biomedical dominance and institutional opacity [35,36,37] and along gendered lines. What this literature has not established is whether these dynamics take the same form in a low-consumption, highly regulated Gulf setting.

There are reasons to expect that they may not. Qualitative research with elderly Arab patients has associated cultural stoicism and religious beliefs with under-reporting of pain, with family support functioning as a principal facilitator of adequate analgesia [38], and culture, religion and language shape pain assessment in Saudi settings [39,40,41]. The same patients are increasingly offered non-opioid alternatives. In this study, “non-opioid drug-delivery systems” refer principally to non-opioid analgesics delivered through skin-applied formats—medicated plasters such as those with lidocaine 5% and high-concentration capsaicin [42,43,44] and topical preparations such as NSAID gels [45]—which act mainly at or near the site of application. These differ from transdermal systems, which are designed to achieve systemic exposure and include opioid patches, so that a skin-applied format is not intrinsically local or non-opioid [46,47]. Oral non-opioid agents such as duloxetine and gabapentinoids have their own evidence base and regulatory position [48,49], and injectable, microneedle, sustained-release and implantable delivery [50,51,52,53,54] and non-pharmacological and digital approaches [55,56,57] lie beyond the study focus. Discrete choice experiments show that patients trade effectiveness against adverse-effect and dependence risk, cost and mode of administration [58,59,60], but they do not explain the reasoning behind these trade-offs and have not been conducted in this region.

Saudi Arabia’s Health Sector Transformation Program under Vision 2030 places patient-centred care and service accessibility at the centre of national reform [61], which makes the patient experience of a restrictive analgesic pathway a policy-relevant question. To our knowledge, no published study has examined how adults living with chronic non-cancer pain in Saudi Arabia experience the opioid access pathway, how stigma and trust operate within it, and how they appraise non-opioid delivery systems. The two questions were examined together because the acceptability of an alternative treatment is unlikely to be independent of the context in which it is offered: trust in the provider is associated with how opioid decisions are received [30,35], and where alternatives are introduced during restriction, patients may appraise them through their experience of that restriction. The aim was to explore the experiences of adults with chronic non-cancer pain in the Al-Ahsa region of Saudi Arabia regarding access to opioid analgesia, stigma and therapeutic trust, and to examine the anticipated acceptability of non-opioid drug-delivery systems and the conditions participants attached to their hypothetical use, with clinician accounts used to contextualise the services within which those experiences occurred. Two research questions guided the work: (i) How do patients with chronic non-cancer pain in Al-Ahsa experience access to, restriction of, and interaction with services around opioid analgesia? (ii) How do they appraise the anticipated acceptability of non-opioid drug-delivery systems as alternatives or adjuncts, and what conditions do they attach to their hypothetical use?

## 2. Materials and Methods

### 2.1. Study Design

A qualitative, multiperspectival interview design was used, analysed through reflexive thematic analysis (RTA) as articulated by Braun and Clarke [62,63]. RTA was selected because the study sought to construct meaning-based patterns across accounts of a morally and politically charged experience rather than to count the frequency of predefined categories; the approach explicitly positions the researcher’s interpretative engagement as a resource rather than a source of bias [63]. A survey or discrete choice experiment could have quantified stated preferences, as prior work has done [58,59,60], but could not have explained how regulatory structures are lived, how stigma is enacted at the counter and the clinic door, or why a patient might refuse an objectively reasonable alternative. The study is described as multiperspectival because, while the patient account is the analytic centre of gravity, a smaller clinician sample was interviewed to contextualise the regulatory and organisational conditions patients described. Clinicians were included because several features of the pathway that patients reported—prescribing rules, dispensing procedures and monitoring alerts—are administered by clinicians and are not directly visible to patients. To keep the analytic focus on patients, themes were developed from the patient corpus, and clinician accounts were read against them to describe the surrounding service conditions, not to judge the accuracy of patient accounts (Section 2.9). The design and reporting are aligned with the Consolidated Criteria for Reporting Qualitative Research (COREQ) [64]; a completed checklist is provided as Supplementary Material S2.

### 2.2. Study Setting

The study was conducted in the Al-Ahsa Governorate of the Eastern Province of Saudi Arabia, one of the Kingdom’s largest governorates by area and home to a population of well over one million distributed between the urban centres of Hofuf and Mubarraz and a wide belt of surrounding villages and agricultural settlements. This distribution is analytically important because participants and clinicians described the pharmacies from which controlled analgesics could be obtained as concentrated in urban facilities, so that distance was a recurrent feature of access in their accounts. The research team did not independently map which facilities dispensed controlled analgesics during the study period; this description reflects participants’ experience rather than a record of dispensing authorisation. Seven settings participated: two governmental tertiary referral hospitals with pain medicine services, one university-affiliated hospital, one private multispecialty hospital, two primary health care centres serving peri-urban and rural catchments, and one hospital-affiliated outpatient pharmacy authorised to dispense controlled analgesics. Together, these settings span the referral pathway that a patient with chronic non-cancer pain in the region would ordinarily traverse, from first presentation in primary care to specialist pain clinic and dispensing. Settings were selected purposively, rather than at random, so that each stage of the regional chronic pain pathway (primary care, specialist pain services and dispensing), each sector (governmental, university and private) and both urban and peri-urban/rural catchments were represented. Community pharmacies outside hospital networks and stand-alone private clinics were not included, and the represented pathway is therefore that of patients in contact with formal hospital and primary care services.

### 2.3. Participants and Sampling

Purposive maximum-variation sampling was used for both samples. For patients, variation was sought across sex, age band, pain condition (musculoskeletal, neuropathic, post-surgical, mixed), duration of pain, current analgesic regimen (currently prescribed an opioid, previously prescribed and tapered or discontinued, never prescribed despite seeking), urban versus rural residence, nationality (Saudi and non-Saudi residents) and payment route (governmental, private and insured). For clinicians, variation was sought across professional group and setting. Recruitment continued until the research team judged that information power was sufficient [65]. Following Malterud and colleagues, the required sample was reasoned from the narrow and specific study aim, the high specificity of the sample (all participants had direct experience of the opioid access pathway, either as recipients of opioid therapy or as patients who had actively sought opioid analgesia from a clinician and been declined or redirected), the use of an established theoretical framework, the strength of dialogue achieved in the interviews and the cross-case analytic strategy [65]; consistent with reflexive thematic analysis, data saturation was not invoked as a stopping rule [66]. In practice, adequacy was judged at analytic meetings held during data generation (approximately after every five patient interviews), at which the team reviewed whether the accumulating interviews offered sufficient depth and variation to address each research question and whether each category of the maximum-variation matrix was represented. Recruitment was closed when the team agreed that recent interviews were elaborating upon rather than extending the analytic dimensions relevant to the aim and that each variation category contained at least one account; this was a reflexive judgement about the sample’s capacity to answer the research questions, not a claim of saturation. The final sample comprised 30 patients (P01–P30) and 12 clinicians (C01–C12). Four additional patients were approached and declined, citing time constraints (n = 3) and unwillingness to discuss medication use (n = 1).

### 2.4. Eligibility Criteria

Patients were eligible if they were (i) aged 18 years or older; (ii) living with non-cancer pain of at least six months’ duration, self-reported and corroborated by a clinical record of a chronic pain diagnosis; (iii) currently receiving, previously prescribed, or having sought pharmacological management for that pain within the Saudi health system; (iv) able to give informed consent; and (v) able to participate in an interview in Arabic or English. Patients were excluded if they had active cancer-related pain, a documented diagnosis of substance use disorder for which they were receiving treatment (in order to keep the analytic focus on non-cancer chronic pain and to avoid conflating two distinct experiences of restriction), acute cognitive impairment, or acute psychological distress that in the judgement of the treating team made participation inadvisable. People receiving treatment for substance use disorder encounter a distinct regulatory and clinical pathway (addiction services, opioid agonist treatment and associated monitoring), so their accounts of restriction would reflect a different system and a different form of stigma; combining the groups risked obscuring the experience of patients whose restriction arises within chronic pain care. The consequence is that the findings do not describe people living with both chronic pain and a treated substance use disorder, in whom stigma and restricted access are likely to be compounded (Section 4.6). Clinicians were eligible if they were licensed physicians, registered nurses or pharmacists with at least twelve months’ experience of managing or dispensing analgesia for chronic non-cancer pain in one of the study settings.

### 2.5. Recruitment Procedure

Recruitment was conducted through the participating services rather than by the research team directly in order to avoid any impression that participation was connected to ongoing prescribing decisions. Clinical staff not involved in the study identified potentially eligible patients from clinic lists, provided the information sheet and asked permission for the research team to make contact. Staff screened clinic lists against the eligibility criteria and the variation categories sought (Section 2.3), and as recruitment progressed the team indicated which categories remained under-represented. Because patients were identified by staff of the services under study, staff may have been more likely to approach patients they regarded as suitable or cooperative and less likely to approach those with strained relationships with the service; this potential gatekeeping is acknowledged as a limitation (Section 4.6). Staff kept a screening log: 47 potentially eligible patients were listed, of whom 13 were not approached (variation category already filled, n = 8; could not be reached, n = 3; judged by the treating team to be acutely unwell, n = 2). The remaining 34 were contacted by the research team; 4 declined (see Section 2.3), and 30 participated. Patient participants were recruited from the seven settings as follows: tertiary hospital A, n = 6; tertiary hospital B, n = 5; university-affiliated hospital, n = 5; private hospital, n = 6; primary health care centre A, n = 3; primary health care centre B, n = 3; authorised outpatient pharmacy, n = 2. Interested patients were then telephoned by a member of the team, given a full explanation and offered at least 48 h to decide. Clinicians were recruited by direct invitation through service leads. It was made explicit in writing and verbally that participation, refusal or withdrawal would have no bearing whatsoever on current or future treatment, prescriptions or employment. No participant had a prior clinical or supervisory relationship with the interviewers.

### 2.6. Data Collection

Data were generated through individual semi-structured interviews conducted between 4 May and 26 June 2026. Patient interviews were conducted by M.S., a PhD-prepared nursing academic/researcher, and clinician interviews were conducted by S.I.A., a physician-researcher. Neither interviewer had clinical or supervisory responsibility for the participants they interviewed. Patients chose the location: 19 interviews took place in a private room within the participating facility, 7 by telephone and 4 in the participant’s home, each at a time of the participant’s choosing. Clinician interviews took place in private offices or by video call. Interviews lasted between 42 and 88 min (patients, mean 61 min) and between 35 and 55 min (clinicians, mean 44 min). Twenty-six patient interviews were conducted wholly in Gulf colloquial Arabic, three in a mixture of Arabic and English, and one in English. Field notes recording setting, non-verbal communication, interruptions and the interviewer’s immediate impressions were written within two hours of each interview and were used analytically. No repeat interviews were conducted, although eight participants were re-contacted by telephone for clarification of specific statements. Interview modality was recorded for every transcript. Because telephone and home interviews may alter rapport, privacy and the availability of non-verbal cues [67], extracts were read with their modality in view, and the team examined whether disclosures of sensitive behaviour, such as concealment or use of a relative’s medication, were confined to particular modalities. No such pattern was identified: telephone interviews (mean 54 min) were somewhat shorter than face-to-face interviews (mean 63 min) but contained comparable disclosures of sensitive behaviour. Modality was not treated as a formal comparison variable.

### 2.7. Interview Guide Development

A semi-structured interview guide was developed from the international literature on opioid access, stigma and trust [25,26,27,35,36], from regional evidence on regulatory barriers [12,13,18], and from the preference literature on analgesic attributes [58,59,60], then adapted for cultural and linguistic appropriateness through review by two bilingual clinicians and one patient adviser living with chronic pain. The guide covered five domains: the pain story and its impact; the pathway to obtaining analgesia; experiences at the point of prescribing and dispensing; trust and the therapeutic relationship; and knowledge of, attitudes towards and conditions for using non-opioid delivery systems. The guide was theoretically informed, and its probes explicitly addressed several topics that later featured in the themes, including distance to dispensing sites, running out of medication, pharmacy registers and supervision, the need to prove legitimate pain, trust in individuals versus institutions, use of relatives’ medicines, decisions presented as external rules, cost and supply continuity, clinician endorsement and compatibility with prayer, ablution and fasting (Supplementary Material S1). Findings on these topics therefore reflect deliberate exploration rather than spontaneous emergence (Section 2.9). To support the fifth domain without leading participants, a neutral one-page visual card depicting generic delivery formats (oral tablet, medicated plaster or patch, topical gel, injectable and implanted device) was offered after participants had first been asked open-ended questions about alternatives. The card, reproduced as Supplementary Material S3, depicted formats rather than specific agents and did not distinguish topical from transdermal systems or opioid from non-opioid patches; participants’ interpretations of these formats are therefore reported as perceptions. The guide was piloted with three patients, after which two questions were reworded: the formal Arabic term for controlled medicines was replaced with the colloquial expressions participants used (for example, “the controlled medicine” or “the medicine with the paper”), and a double-barrelled question on prescribing and dispensing was split into two. Because these changes altered wording but not the topics covered or the order of domains, the pilot interviews addressed the same content as subsequent interviews and were retained in the dataset; consistent with reflexive thematic analysis, consistency was sought in the topics explored rather than in identical wording. The full guide is provided as Supplementary Material S1. It is reproduced exactly as used during data collection and therefore retains the study’s original working title, which referred to “Preferences for Non-Opioid Drug Delivery Systems”; the article title was revised during peer review to reflect the anticipated, hypothetical nature of the acceptability data.

### 2.8. Transcription and Translation

Interviews were audio-recorded with written consent and transcribed verbatim in the language of the interview by a bilingual member of the research team. Transcripts were checked against the recordings in full by a second bilingual researcher, and discrepancies were resolved by listening jointly to the original audio. Analysis was conducted on the Arabic transcripts to preserve idiom and register. Only quotations selected for publication were translated into English, using forward translation by one bilingual researcher, independent back-translation by a second, and reconciliation of discrepancies by discussion with reference to the original recording. Where an Arabic idiom carried meaning that a literal translation would lose, an explanatory note is provided in square brackets within the quotation. Translation aimed at conceptual rather than literal equivalence [68]: translators were native speakers of Gulf Arabic, and colloquial expressions were rendered to preserve their local meaning and glossed where necessary. For example, participants used al-waraqa (“the paper”) for a prescription or authorisation; because the term is used colloquially for official documents irrespective of format, quotations using it are not taken as evidence that paper prescriptions were in use at the time of interview (Section 3.3). Transcripts were not returned to participants for correction, as pilot feedback indicated that this would be burdensome and might inhibit candour; instead, the interviewer summarised his understanding of key points at the end of each interview for the participant to confirm or correct, and eight participants were re-contacted to clarify specific statements. These procedures supported immediate checking of meaning; participants did not review the developed themes or analytic findings [69].

### 2.9. Data Analysis

Analysis followed the six phases of reflexive thematic analysis [62,63], conducted at a latent level with a combined orientation: coding remained open to meanings not anticipated by the interview guide, but data generation had been deliberately structured by theoretically informed probes (Section 2.7), and the analysis was sensitised to the concepts of stigma [27] and epistemic injustice [32,34]. The topics that make up most sub-themes were therefore explored directly through the interview guide; what the analysis generated was the organisation of these topics into themes, the meanings participants attached to them and the interpretive links between them. Findings are accordingly presented as participants’ responses to topics the interview explored, except where noted. In phase one, two researchers immersed themselves in the data by repeated listening and reading, recording initial analytic notes. In phase two, the full dataset was coded line by line in NVivo 14, generating 412 initial codes across the patient corpus and 96 across the clinician corpus; coding was carried out independently by two researchers for the first eight transcripts and then collaboratively, with divergence treated as an analytic resource to be discussed rather than an error to be reconciled into a reliability coefficient [63]. When the two coders read an extract differently—for example, whether an account of taking fewer tablets expressed cultural endurance or self-rationing in response to supply—both readings were recorded in an analytic memo and discussed with reference to the full transcript and field notes. Disagreements were resolved not by vote but by adopting the reading best supported across the dataset, and unresolved alternatives were carried forward as questions for theme review; the discussion of endurance and constraint informed the framing of sub-theme 1.2. In phase three, codes were clustered into candidate themes using visual mapping. In phase four, candidate themes were reviewed against coded extracts and against the entire dataset; two initial candidate themes—one on financial burden and one on family involvement—were dissolved and redistributed, as they proved to cut across the other themes rather than stand alone. In phase five, themes were defined, named and given the participant-derived phrases that title them here. In phase six, the report was produced, with extracts selected for their analytic contribution and for representation across the sample. Patient and clinician datasets were coded separately and integrated at phase four. Integration proceeded in one direction: candidate themes were developed from the patient corpus, and clinician codes were then mapped onto them to identify where clinician accounts described the service conditions behind a patient experience, where they offered a different attribution for the same event, and where they diverged. Clinician accounts were not used to confirm or refute patient accounts, no theme was built from clinician data alone, and points of divergence are reported in Section 3.7. No formal cross-case comparison by sex, age, residence or treatment status was undertaken; where the Results locate an experience in a subgroup, this indicates whose accounts it appeared in, not its relative prevalence, and words such as “several” or “some” are descriptive rather than counts.

### 2.10. Rigour and Reflexivity

Trustworthiness was addressed against the criteria of Lincoln and Guba [70]. Credibility was supported by data generation across seven settings over approximately seven weeks, in-depth interviews supplemented by field notes, the inclusion of clinician perspectives, independent double coding of early transcripts, discussion of divergent readings, and in-interview summary checking with clarification calls (Section 2.8); participants did not review the final themes. Transferability is supported by the contextual description of the setting, sampling frame and participants (Section 2.2, Table 1). Dependability was addressed through an audit trail comprising the interview guide, field notes, coding framework versions, thematic maps and dated analytic memos, and confirmability was addressed by grounding claims in extracts and by the reflexive journal described below.

Reflexivity was treated as an ongoing practice across personal, interpersonal, methodological and contextual dimensions rather than a single positioning statement M.S. is a male PhD-prepared nursing academic/researcher with a longstanding programme of qualitative research on pain and ageing in Arab populations, including prior work on barriers to pain management in elderly Arab patients [38]; this background sensitised the team to cultural stoicism and family mediation, and the reflexive journal was used specifically to interrogate the risk of hearing endurance where participants were describing constraint. S.I.A. is a physician–researcher based in Al-Ahsa whose familiarity with the regional service configuration aided contextual interpretation but raised the possibility of insider assumptions; he conducted clinician interviews without clinical or supervisory responsibility for participants. R.M.A., based in pharmaceutical sciences, contributed pharmacological interpretation. Across the team, professional positions could predispose interpretation towards clinician- or system-centred readings; analytic memos therefore documented instances in which initial codes were revisited when the team judged that they had adopted the system’s framing of the patient rather than the patient’s own.

### 2.11. Ethical Considerations

The study was conducted in accordance with the Declaration of Helsinki and was approved by the Research Ethics Committee of King Faisal University, Al-Ahsa, Saudi Arabia (Approval No. ETHICSA-2026-2525; approval date: 1 May 2026). Institutional research permissions were additionally obtained from each participating facility. All participants provided written informed consent prior to data collection, including specific consent for audio-recording and for the publication of anonymised verbatim quotations; for the two participants who were unable to read, the information sheet was read aloud in full in the presence of an impartial witness who countersigned the consent form. Participants were informed of their right to withdraw at any point without giving a reason and without any consequence for their care. Participants received no payment for participation; patients who travelled specifically to attend an interview were reimbursed SAR 100 for transport costs. Because the interviews addressed a topic that participants might reasonably fear could affect their prescriptions, particular care was taken to separate the research from the clinical encounter, as described in Section 2.5. Identifiers were removed at transcription and replaced with participant codes; audio files were stored separately from transcripts on an encrypted, password-protected institutional server accessible only to the research team. Because participants described sensitive behaviours within a geographically defined health system, the risk of deductive disclosure was assessed when quotations were selected and when descriptors were revised for this version [71]. Descriptors were limited to participant code, sex and age band; exact age, residence, pain diagnosis and treatment pathway were removed; clinician descriptors were limited to professional group; and details within quotations that could identify a participant, such as occupation, were replaced with bracketed general terms. A distress protocol was in place, under which the interview would be paused and, with the participant’s agreement, a referral offered to the treating service; this was activated on two occasions, both of which concluded with the participant choosing to continue.

## 3. Results

### 3.1. Participant Characteristics

Thirty patients and twelve clinicians participated. The patient sample was evenly balanced by sex, spanned five decades of age and four broad pain categories, and included participants at every point of the analgesic pathway: currently prescribed an opioid, tapered or discontinued, and never prescribed despite having sought pharmacological relief. Just over a third lived outside the two main urban centres. The median duration of chronic pain was 6 years (range 1–24 years). Participant characteristics are summarised in Table 1.

Table 1 indicates that each maximum-variation category was represented, although some cells were small (for example, participants aged ≥65 years, n = 5, and insured participants, n = 5), so the accounts of these groups rest on fewer voices. Among the 21 participants with current or past opioid treatment, the opioid analgesics involved were tramadol (n = 12), codeine-containing combinations (n = 4), oral morphine (n = 2), transdermal fentanyl (n = 2) and oxycodone (n = 1), with a treatment duration of a median of 26 months (range 4–118 months); treatment was taken regularly by 13 and intermittently or as needed by 8 participants. The nine participants never prescribed an opioid had each asked for or been referred for opioid analgesia (tramadol, n = 6; a codeine-containing combination, n = 2; an unspecified “stronger painkiller”, n = 1) and had been declined or redirected to other treatment; their direct experience of the pathway therefore consisted of referral, consultation and refusal encounters rather than dispensing or tapering. Several participants also took, or had taken, other controlled medicines, chiefly gabapentinoids; accounts concerning these medicines (from 9 participants) were coded separately, and the opioid-related findings are based on accounts of opioid analgesics.

### 3.2. Overview of Themes

**Figure 1:**
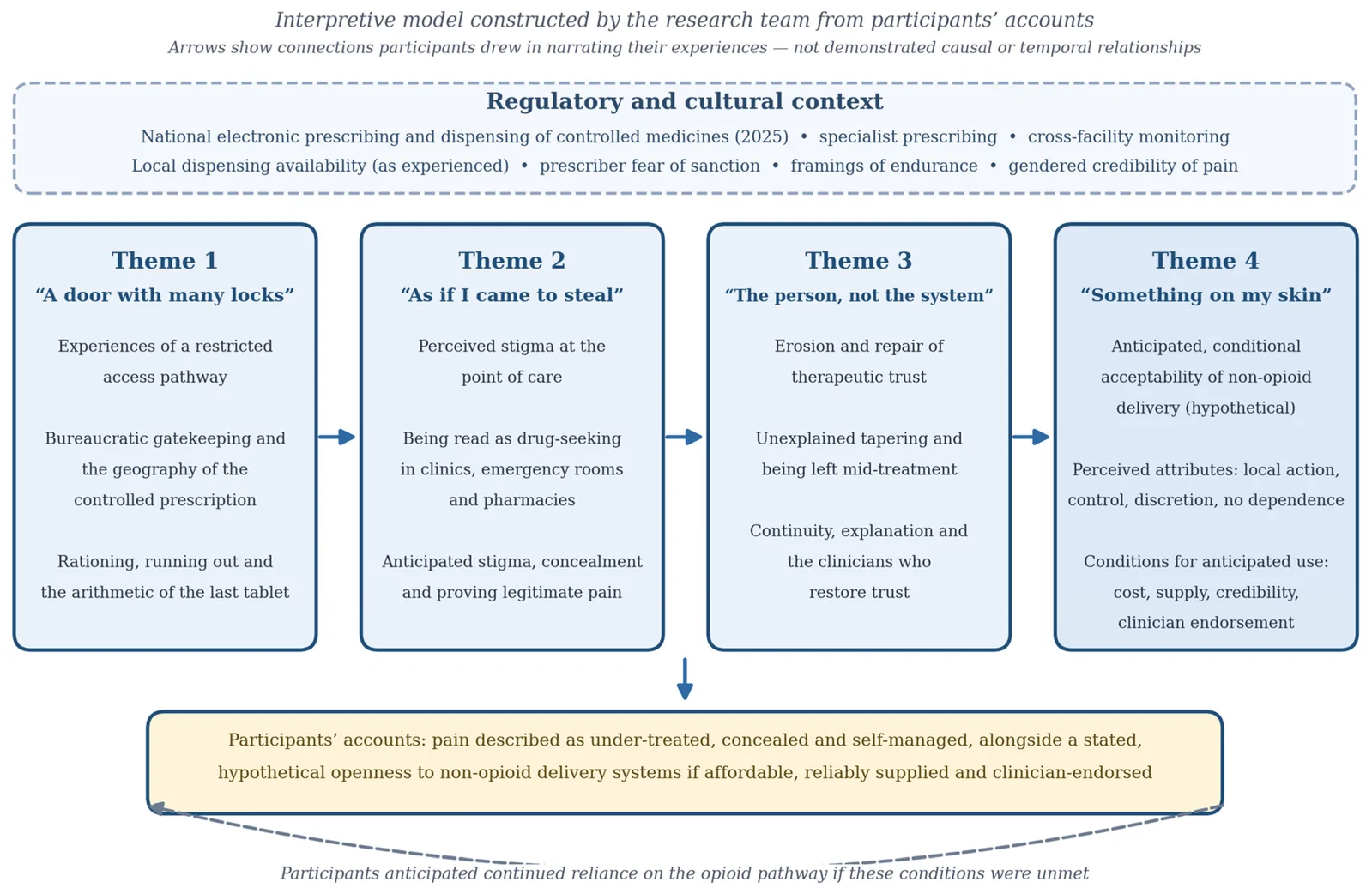
Interpretive model, constructed by the research team from participants’ accounts, of the relationships between the four themes. Arrows indicate connections that participants drew when narrating their experiences; they do not represent demonstrated causal or temporal relationships, which a cross-sectional qualitative design cannot establish. The dashed arrow represents participants’ anticipation that, if the conditions they described were unmet, they would continue to rely on the opioid pathway.

Four themes were constructed from the dataset, each comprising two sub-themes. The research team interpreted the themes as related rather than separate: in participants’ accounts, experiences of restricted access were narrated alongside encounters in which they felt treated as suspects; participants linked these encounters to distrust of institutions while retaining trust in particular clinicians; and they drew on these experiences when appraising non-opioid delivery systems, towards which they expressed a conditional, sceptical and hypothetical openness. The themes and sub-themes are summarised in Table 2, and the team’s interpretive model of their relationships is shown in Figure 1.

### 3.3. Theme 1: “A Door with Many Locks”—Navigating a Restricted Access Pathway

Participants did not describe opioid analgesia primarily as a drug. They described it as a procedure: a sequence of referrals, signatures, journeys and time windows that had to be completed correctly, in pain, and repeated. What made this theme distinctive was not that access was difficult but that patients had become expert in the difficulty—able to recount the sequence, its failure points and its unwritten rules with a precision that reflected years of repetition. Clinicians described the same architecture from the other side, as a set of rules they administered but had not designed and could not relax.

Participants’ accounts in this theme span different periods. With a median pain duration of six years, many described experiences from before the national transition to electronic prescribing and dispensing of controlled medicines in 2025 [19,72], while others described the electronic system as they encountered it at the time of interview. The features they described also operate at different levels: specialist prescribing and cross-facility visibility of dispensing are features of the national system [72], whereas the local availability of pharmacies able to dispense controlled analgesics, appointment waits and pharmacy procedures reflect local implementation and participants’ experience of it. When participants spoke of “the paper”, they referred to the prescription or authorisation (Section 2.8). Facility-level implementation of the electronic system was not documented, and no claim is made that paper-based processes persisted in the participating facilities in 2026.

#### 3.3.1. Sub-Theme 1.1: Bureaucratic Gatekeeping and the Geography of the Controlled Prescription

A recurrent feature of participants’ accounts was that the pathway could not be shortened. A prescription for a controlled analgesic required a specialist, the specialist required a referral, the referral required an appointment, and participants described the resulting prescription as obtainable only at particular locations and within a limited window. Participants living outside Hofuf and Mubarraz described this as turning a clinical matter into a transport problem.

*“The medicine is not far. The paper is far. I go to the health centre, they say we cannot write this, you need the pain doctor. The pain doctor is in the city, and the appointment is after five weeks. Then the paper is in my hand and I must go back to the city again, because our pharmacy is not allowed. Three journeys for one box. And all of this while my back is what it is.”*

(P07, male, 50–64 years)

Variants of the phrase “the paper is far” appeared in the accounts of several rural participants and capture the analytic point: as participants described it, the scarce resource was not the medicine but the authorisation.

Clinicians described the same features from the prescriber’s side, locating them in regulation rather than in clinical judgement and acknowledging that the burden fell on patients.

*“Everything they complain about is real. I cannot delegate the prescription, I cannot give three months, the pharmacy that can dispense it is not in their village. I am not defending the system to you. But if I bend one rule, it is my licence. So what happens is the patient carries the whole weight of a policy that was written for a different problem.”*

(C03, pain medicine physician)

As participants described it, no individual within the pathway refused them; refusal was distributed across referral rules, dispensing geography and appointment supply, which participants found hard to contest and experienced as impersonal indifference.

#### 3.3.2. Sub-Theme 1.2: Rationing, Running out and the Arithmetic of the Last Tablet

Participants described continuous self-rationing, which they explained by reference to fixed quantities, the absence of refills and uncertainty about the next authorisation. They presented this not as non-adherence but as prudence in the face of an unreliable supply, and several performed the arithmetic aloud during the interview.

*“I have eleven left and nineteen days. So it is one every second day, and on the day I have none I stay in the bed and I do not go to my son’s house. The doctor tells me take it as prescribed. I want to ask him: with what? Should I take it as prescribed for eleven days and then be an animal for eight?”*

(P21, male, 35–49 years)

Participants described consequences of rationing that extended beyond pain control, including declining work shifts, missing family obligations and withdrawing from social and religious gatherings in order to preserve medication for days they judged unavoidable so that, in their accounts, restriction reorganised social participation itself.

*“I did not go to my brother’s wedding. Not because of the pain—because of the tablets. I calculated that if I go, I will need two extra, and then at the end of the month I will have nothing at all. So I stayed home and I told them I had fever. This is what it makes you: a person who lies to his family about a wedding.”*

(P09, male, 35–49 years)

Other participants described the opposite strategy—consuming the supply early and then enduring an unmedicated interval—and some described this with shame. A dispensing pharmacist described how the same behaviour appears from behind the counter.

*“When someone comes early, my system flags it. What I see is “early request”. What I do not see is that his appointment was cancelled, or that he gave two tablets to his wife who also has pain, or that he took extra during the week he had to travel. The flag does not have a place for any of that. And once it is flagged, the conversation changes completely.”*

(C11, dispensing pharmacist)

In participants’ accounts, a supply intended to be safe was experienced as one that could not be relied upon, and the adaptations they described, such as early requests and irregular use, resembled the behaviours that the pharmacist described the monitoring system as flagging.

### 3.4. Theme 2: “As if I Came to Steal”—Stigma at the Point of Care

Where Theme 1 described a structure, Theme 2 describes an encounter. Participants recounted moments—at a triage desk, a consultation, a pharmacy counter—in which they understood themselves to have been re-categorised from patient to suspect. These moments were remembered with unusual precision, often years later, and were narrated in the idiom of accusation and theft rather than of clinical disagreement.

#### 3.4.1. Sub-Theme 2.1: Being Read as Drug-Seeking in Clinics, Emergency Rooms and Pharmacies

Suspicion was rarely stated explicitly. Participants described inferring it from the sequence and register of questions, from repeated verification of documents, from delay, and from non-verbal conduct—being spoken about rather than to or being made to wait in view of others while a prescription was checked.

*“Nobody said the word. But the questions changed. First it was where is the pain, then it was how long, and then suddenly it was who else writes for you, and do you have this in another hospital, and can I see your ID again. I am a [professional who assesses others], I know when I am being examined. They look at me as if I came to steal something from them.”*

(P02, male, 35–49 years)

Participants who had attended the emergency department described it as especially hostile, relating this to presenting there when their supply had run out and to the absence of an established relationship.

*“In the emergency at three in the morning you are not a patient with a disease, you are a file with a red mark. The doctor did not sit. He stood by the door the whole time. Standing means: you will not be here long, and I have already decided.”*

(P26, female, 18–34 years)

Participants described the pharmacy counter as exposing because it was public; several described visible elements of the transaction—a separate register, an additional signature, the summoning of a supervisor—as an announcement to everyone in the queue. These accounts describe pharmacy procedures as participants encountered them, including before the 2025 transition, rather than current national dispensing requirements.

*“There is a special book for this medicine. He brings it out and everyone in the line sees him bring it out. My neighbour was behind me. She did not say anything, but I know what she saw. After that I started going to a pharmacy far from my district where nobody knows me.”*

(P17, female, 50–64 years)

Clinicians did not deny that suspicion was enacted, but they attributed it differently: whereas patients experienced it as a judgement about them, several clinicians described it as an occupational reflex that they associated with monitoring systems and fear of sanction.

*“If I am honest, my first question now is a screening question, not a clinical one. That is what the system has trained into me. And the patient feels it immediately—they feel that I am checking them, not treating them. I know I am doing it and I still do it, because the risk to me of the other mistake is much bigger.”*

(C06, family physician)

#### 3.4.2. Sub-Theme 2.2: Anticipated Stigma, Concealment and Proving Legitimate Pain

Having been read as suspect once, participants organised subsequent encounters to prevent it. This anticipatory work took two forms: concealment of medication use from social networks, and the assembly of evidence to establish legitimacy in clinical ones. Both were described as exhausting, and both were undertaken by people already depleted by pain.

*“I carry the reports with me. The MRI, the operation paper, the old prescriptions, all in a plastic folder. Before I even sit down I put the folder on the desk. My wife laughs at me. But it is my proof that I am not what they think. A healthy person does not need to carry proof that he is sick.”*

(P11, male, 50–64 years)

Concealment extended into the family and the workplace. Participants described hiding medication in the house, refusing to let adult children collect prescriptions on their behalf, and inventing alternative explanations for absence—reproducing, in private life, the suspicion they had met in clinical life.

*“My husband knows. Nobody else. Not my sisters, not my mother. In our families, if they hear this medicine, immediately the talk starts—is she dependent, what will happen to the children. So I keep it in a box with the sewing things. Twelve years, and I keep my medicine with the sewing things.”*

(P05, female, 35–49 years)

Some women and some younger men described an additional credibility burden. Several women reported that their pain had been attributed to emotional or reproductive causes before physical ones, and some younger men reported feeling screened for recreational motives, which they contrasted with how they believed older patients were treated. These are participants’ reported experiences and perceptions; the study was not designed to compare groups.

*“Every time, the first suggestion is that I am tense, or it is hormonal, or I should rest more. If a man of fifty comes with this same back, do they tell him it is his nerves? I have stopped saying that I am tired, because tired becomes depressed in the file, and depressed becomes not real pain.”*

(P23, female, 35–49 years)

*“I am twenty-nine. That is the whole problem. Because I am twenty-nine, the first thing in his mind is that I want it for something else. An old man with the same pain gets sympathy. I get an investigation.”*

(P30, male, 18–34 years)

One nurse participant described a similar asymmetry from within the service, suggesting that the informal category of the “good patient” rested on characteristics unrelated to pain severity.

*“We say it among ourselves without noticing—this one is genuine, that one is doubtful. And if you examine what makes someone genuine, it is that they are older, quiet, they come with a relative, they do not ask for a specific medicine by name. It is not the pain that decides. It is the manner.”*

(C08, registered nurse)

### 3.5. Theme 3: “I Trust the Person, Not the System”—Erosion and Repair of Therapeutic Trust

The third theme concerns trust. Many participants distinguished between confidence in individual clinicians, which was often high, and confidence in the institution, which was often absent, and they connected this distinction to what they chose to disclose and how they used their medicines.

#### 3.5.1. Sub-Theme 3.1: Unexplained Tapering and the Experience of Being Left Mid-Treatment

Ten participants had experienced dose reduction or discontinuation in the preceding two years. In their accounts, how the reduction was experienced depended less on the reduction itself than on whether it had been explained and whether anything had been offered in its place. Where a taper was communicated as an external rule—a new policy, a circular, an unnamed authority—participants described it as abandonment.

*“He did not say I think it is better for your health. He said there are new instructions. Instructions from where? From whom? I have been his patient for six years and suddenly I am talking to a messenger. If it is bad for me, tell me it is bad for me and I will accept. But do not hide behind a paper.”*

(P19, male, 50–64 years)

Several participants described the reduction as arriving without any accompanying plan, which they experienced as a withdrawal of care.

*“They reduced and then there was nothing. No other medicine, no physiotherapy appointment, no follow-up. Just less. If you take away the ladder you should at least show me another way down. Otherwise it is not treatment, it is only subtraction.”*

(P13, female, 35–49 years)

Participants described responding with concealment and self-management rather than confrontation, including obtaining analgesia from relatives, purchasing non-prescription preparations in larger quantities, using medication prescribed for a family member, and in several accounts, ceasing to report the true severity of their pain, on the reasoning that honesty had become risky.

*“Now when he asks me the number from ten, I say six. Always six. If I say nine he becomes worried and he reduces more, or he sends me for more tests, or he writes something in the file. Six is safe. So he is treating a six that does not exist, and I am living with a nine.”*

(P08, male, 50–64 years)

#### 3.5.2. Sub-Theme 3.2: Continuity, Explanation and the Clinicians Who Restore Trust

Against this, participants described specific clinicians whose conduct had restored something. The elements they described were modest and recurred across accounts: continuity of the same person over time, an explanation that named the constraint honestly, an acknowledgement that the constraint was unjust or at least regrettable, and a concrete plan that survived the consultation.

*“Doctor A. is the only one who told me the truth. He said: I am limited, this is what I am allowed, I am not able to give you more and I am sorry for that, but here is what we will do instead and I will see you myself in three weeks. He did not fix my pain. But I stopped feeling that I was fighting him.”*

(P04, female, 50–64 years)

Participants attached this trust to persons rather than institutions and were explicit that it did not generalise. Several described following a particular clinician between facilities and expressed anxiety about that clinician’s leave or transfer.

*“If they move him, I am finished. Not because there is no other doctor. Because I will have to explain myself again from the beginning to a person who does not know me, and be looked at again in that way. I trust the person, not the system. The system I do not know at all.”*

(P25, male, 35–49 years)

Several participants identified nurses as the people who provided continuity, particularly by explaining what had been decided and following up between appointments. Nurse participants described this role from their side and connected it to the limited formal authority nurses hold in the pathway.

*“The nurse is the one who calls, who explains what the doctor meant, who notices that you did not come. The doctor gives you eight minutes. She is the one who makes it a treatment and not a transaction.”*

(P22, female, ≥65 years)

*“We are the continuity in this pathway, but we have no authority in it. I can see that a patient is deteriorating and is going to run out, and all I can do is try to get him an earlier appointment. Give the nurse a defined role in this pathway and half of these problems disappear.”*

(C02, registered nurse)

This nurse’s expectation that a defined role would resolve “half of these problems” was not borne out across the dataset: patients’ accounts of distance, supply and cost (Themes 1 and 4) described constraints outside any clinician’s control. It is therefore reported as a clinician’s view rather than as a finding.

Clinicians’ own constraint was visible to patients and was interpreted generously by some and bitterly by others; either way, patients understood that the person in front of them was also constrained.

*“I do not blame him personally. He is also afraid. You can see it—when he writes this prescription his hand is different from when he writes for blood pressure. We are two people afraid of the same paper.”*

(P28, male, 50–64 years)

### 3.6. Theme 4: “Something I Can Put on My Skin”—Anticipated Acceptability and Conditions for Hypothetical Use of Non-Opioid Drug Delivery Systems

The final theme addresses the second research question. Because most participants had not used the discussed formats and responses were elicited partly with a visual card (Supplementary Material S3), this theme reports anticipated acceptability and stated conditions for hypothetical use, not treatment choices or actual uptake. Participants expressed more openness to non-opioid delivery systems than the team had anticipated, but this openness was conditional, and their reasoning drew on the experiences described in Themes 1 to 3.

#### 3.6.1. Sub-Theme 4.1: Valued Attributes—Local Action, Self-Control, Discretion and Freedom from Dependence

Participants described four valued attributes. The first was locality: participants found it intuitively persuasive that a medicine should be applied where the pain is and contrasted this favourably with the perceived indiscriminate reach of an oral drug. This was a perception: participants generally assumed that anything applied to the skin acts only where it is applied, which holds for some topical products but not for transdermal systems designed for systemic absorption (Section 4.4).

*“The tablet goes to the whole body to treat one place in the back. It passes the stomach, the liver, the head, everything. If there is something I can put on my skin, on the place itself, this is more reasonable to me. Treat the place that is complaining.”*

(P16, male, 35–49 years)

The second was control. Because a patch or gel could be applied and removed by the patient, it promised to restore a small domain of autonomy to people whose accounts otherwise described complete dependence on institutional permission.

*“The plaster I put on myself. I decide the day, I decide the hour, and if I do not want it I take it off. Do you understand what this means for me? For eight years, somebody else has decided everything about my medicine. This one thing would be mine.”*

(P12, female, 35–49 years)

The third was discretion. Formats that attracted no special register, no supervisor and no separate book were valued in the light of the pharmacy experiences described in sub-theme 2.1; the participant quoted there (P17) described being able to buy a cream unobserved as “a small thing and … not a small thing”.

The fourth was perceived freedom from dependence and its moral weight. Participants framed non-opioid formats as morally lighter, and several connected this explicitly to religious and familial concern about intoxicants and about becoming a burden.

*“With this medicine there is always the question in your head and in your family’s head: will I become attached to it. With the plaster, this question does not exist. There is nothing to be attached to. My mother would not lie awake about a plaster.”*

(P09, male, 35–49 years)

Participants’ belief that a plaster carries no dependence potential is reported as a perception; whether a skin-applied product carries such a risk depends on the agent it delivers rather than on the format (Section 4.4).

The valued and rejected attributes reported across the sample are summarised in Table 3.

#### 3.6.2. Sub-Theme 4.2: Conditions for Anticipated Use—Cost, Supply, Credibility and Clinician Endorsement

Stated openness is not the same as use. Participants set out conditions for considering these formats and related several of them to earlier disappointments. Cost was a prominent concern, particularly for participants accustomed to governmental provision, for whom a shift to a format purchased out of pocket represented a transfer of the burden rather than a solution.

*“The tablet from the government pharmacy costs me nothing except my dignity. The cream from outside costs one hundred and something, every month, forever. So which one is really cheaper for a man who is not working because of his back? If they want us to use it, it must be in the list.”*

(P21, male, 35–49 years)

Reliable supply was a second condition, which participants related to the rationing experiences described in sub-theme 1.2. Participants were unwilling to reorganise their pain management around a product that might disappear.

*“I will not build my life again on something that is available today and finished tomorrow. I have already had that. Bring it to the pharmacy in my district, keep it there for a year, and then I will believe it exists.”*

(P07, male, 50–64 years)

Credibility for severe pain was a third condition. Participants said they would accept non-opioid delivery systems as adjuncts or for mild to moderate pain but were sceptical about them for crisis pain, and several articulated an explicit fear that the alternatives were being introduced as a means of closing the opioid door altogether.

*“For the daily pain, yes, I accept it, I will try anything. But when the pain comes at two in the morning and I cannot breathe from it, will a sticker help me? Be honest with me. And I am afraid that they will give me the sticker so that they can say: you see, he has a treatment, he does not need the other one.”*

(P28, male, 50–64 years)

Cultural and devotional fit formed a fourth condition. Compatibility with prayer, ablution and fasting was an interview probe (Supplementary Material S1, Question 20), so this condition is reported as a response to a topic the interview explored rather than as a spontaneously raised concern; participants who discussed it wanted explicit guidance rather than reassurance.

*“Nobody could tell me clearly: with this plaster on my arm, is my ablution correct? And in Ramadan, is there any problem? These are not small questions for me. I asked the pharmacist and he said he thinks it is fine. He thinks. I need someone to know.”*

(P14, female, 50–64 years)

A final condition, emphasised by many participants, was endorsement by a trusted clinician. Participants distinguished sharply between an alternative recommended within a continuing relationship and one offered at the point of refusal, and one clinician described the timing of the offer as shaping its reception.

*“If Doctor A. tells me try this, I try it that same day. If a stranger tells me when he is refusing my medicine, then it is not advice, it is a consolation prize. The same product. Completely different thing.”*

(P04, female, 50–64 years)

*“We make a mistake in the timing. We offer the alternative in the same sentence in which we reduce the opioid, and then the alternative is contaminated by the reduction. It should be introduced weeks earlier, by someone the patient trusts, as an addition. Otherwise you have given them a good treatment and guaranteed they will not use it.”*

(C03, pain medicine physician)

### 3.7. Integrative Summary Across Themes

Read together, the four themes suggest to the research team an interpretive account in which participants’ experiences were connected rather than independent. Participants described scarcity and unpredictability of supply (Theme 1) alongside adaptive behaviours—early requests, dose-splitting and attendance at unfamiliar services—that the pharmacist participant described as the behaviours monitoring systems flag. They also described being treated as suspects at the point of care (Theme 2), and several linked these encounters to a reluctance to be candid and to distrust of institutions, while trust persisted in particular named individuals (Theme 3). When appraising non-opioid delivery systems (Theme 4), participants drew on these experiences: the attributes they valued—local action, control, privacy and absence of moral weight—corresponded to aspects of their situation they wished to change, and they explained their refusal to regard alternatives as replacements by reference to earlier, unexplained withdrawals of relief. The conditions they set can therefore be read as describing the trust participants considered necessary before an alternative would be credible. This sequence is the research team’s interpretation of participants’ accounts (Figure 1); the study was not designed to establish the direction, ordering or causal nature of these relationships, which may be reciprocal or shaped by factors not captured here.

Clinician accounts were broadly consistent with patients’ descriptions of the structural features of the pathway, and no clinician disputed that restriction and suspicion occurred. The two corpora diverged, however, in attribution and emphasis. Patients tended to experience suspicion as a judgement about their character, whereas clinicians described it as a reflex associated with monitoring and fear of sanction (sub-theme 2.1); and a nurse participant’s view that a defined nursing role would resolve many problems contrasted with patients’ accounts of distance, supply and cost that no clinician role could address (sub-theme 3.2). Two clinicians offered accounts that challenged a patient-centred reading of the pathway. A pain medicine physician (C01) described patients who had obtained prescriptions from more than one facility before cross-facility monitoring made this visible, and regarded that monitoring as clinically justified; a dispensing pharmacist (C12) described the separate register as protecting patients as well as staff. These accounts do not contradict patients’ descriptions of their experience, but they show that some clinicians regarded as legitimate safeguards features that patients experienced as stigmatising, and they informed the interpretive rather than causal framing of Figure 1.

## 4. Discussion

This study is, to our knowledge, the first to examine how adults living with chronic non-cancer pain in Saudi Arabia experience a restrictive opioid regulatory environment and the first anywhere in the Gulf to connect that experience to the anticipated acceptability of non-opioid drug-delivery systems. Its central interpretation is that participants described restriction less in terms of dose than in terms of relationship: a pathway they felt treated them as a risk to be managed, encounters in which they felt recategorised as suspects, and a distinction between trust in individuals and distrust of institutions on which they drew when appraising alternatives. This matters because it concerns aspects of policy that prescribing statistics do not capture.

### 4.1. Access as Experienced Within a Changing Regulatory Architecture

The features participants described—specialist prescribing; fixed quantities without refills; and, in their experience, a limited number of dispensing points—correspond to regulatory features documented across 19 Arab countries [12] and to the physician hesitancy and treatment delay reported after gabapentinoid reclassification in Saudi Arabia [13]. They should, however, be read against the national transition to electronic prescribing and dispensing announced in February 2025 [19,72]. Specialist prescribing and cross-facility visibility of dispensing are features of the current national system [72]; paper documents and pharmacy registers appeared in accounts that spanned the period before the transition; and the local availability of dispensing pharmacies reflects implementation rather than national rule. Electronic prescribing may ease some burdens participants described—for example, by making prescriptions available digitally [19]—while leaving others, such as specialist referral and appointment supply, unchanged; whether it alters the experiences reported here requires further study. What this study adds is an account of how such an architecture was experienced by patients in a low-consumption Gulf setting: as a logistical project whose costs, participants reported, fell most heavily on those living far from urban services. Distance to dispensing points was prominent in participants’ accounts of obtaining relief, echoing the supply and regulatory barriers identified at health-worker level in the context of cancer pain [11] and illustrating how comparable barriers may be experienced by patients as journeys undertaken in pain. These findings require careful interpretation. Arguments that restriction designed for high-consumption crises may be poorly calibrated for low-consumption settings, and that under-treatment rather than excess is the dominant risk [7,8,9,10], concern principally access to opioids where they are indicated, such as cancer and palliative pain. For chronic non-cancer pain, the uncertain long-term benefit and dose-related harms of opioid therapy [14,15,16] mean that restriction, tapering or a decision not to prescribe may often be clinically appropriate. Our data do not suggest that more opioids should have been prescribed to these participants, and we did not assess the clinical appropriateness of individual prescribing decisions. They suggest instead that the way restriction was organised and communicated—distance, unexplained tapering and public marking—was central to participants’ accounts of their experiences, independently of whether the underlying clinical decision was sound. The rationing behaviours participants described—splitting doses, stockpiling and requesting early—also resemble signals that, according to the pharmacist participant, monitoring systems flag as risk. We offer this as an interpretive proposition rather than a demonstrated mechanism: adaptations to an uncertain supply may be read as indicators of risk, a possibility that could be examined with linked prescribing and dispensing data.

### 4.2. Stigma and Epistemic Injustice at the Point of Care

The accounts in Theme 2 closely parallel Dassieu and colleagues’ Canadian findings that patients are labelled as drug-seeking, particularly in emergency departments, and that established relationships buffer that labelling [25], as well as their documentation of concealment and self-advocacy in everyday social interaction [26]. They are also consistent with the multi-level model of opioid stigma proposed by Bulls and colleagues [27] and with evidence that stigma is perceived on both sides of the encounter [29]. The parallel with Nelson’s Nigerian study is particularly instructive: there, stigma operated through disciplinary prescribing and dispensing practices that categorised patients as irresponsible and pushed them towards herbal alternatives [28]; here, participants described responding with concealment, use of distant pharmacies and non-disclosure. That similar dynamics are described in two settings with different opioid-use profiles raises the possibility that they are associated with control arrangements rather than with any particular national crisis, a proposition that comparative research could test.

Our data may extend this literature in two ways. First, participants described the pharmacy counter as a site of enacted stigma, in which visible elements of control—a separate register, a countersignature, and a supervisor summoned in view of a queue—were experienced as public identification. Because these accounts spanned the period before and after the 2025 transition, we cannot say whether electronic dispensing has changed such procedures; where they persist, they carry an unintended social meaning and may be amenable to redesign. Second, the credibility asymmetries that some participants reported by gender and age are consistent with international evidence on the dismissal and minimisation of women’s pain [40,41] and were articulated through a local repertoire, in which some women described their pain being attributed to emotional or reproductive causes and some young men described their requests being read as recreational. The nurse participant’s account of how the informal category of the “genuine” patient is assembled from demeanour rather than pathology illustrates the mechanisms of testimonial injustice described by Côté [32,34] and the pathocentric epistemic injustice examined by Charette [33]. A contribution specific to the Saudi context follows: where cultural expectations of stoicism have been associated with under-reporting of pain [38,39], participants described a service environment in which emphatic reporting could attract suspicion, leaving them little room to be heard as credible in either register.

### 4.3. Trust: Personal Repair Against Institutional Erosion

The distinction participants drew between trusting a person and trusting a system is consistent with evidence that shared decision-making is associated with less opioid misuse, partly through its effect on trust in the provider [30], and with findings that patients’ experience of opioid management turns on expectation and communication [31]. Our tapering findings align closely with Kosakowski and colleagues, whose participants experienced reductions as inadequately justified and harmful, with relationship quality associated with how reductions were interpreted [35], and with the divergence Nevedal and colleagues identified between patients who emphasise the benefits of continuing and providers who emphasise the benefits of stopping [36]. Participants here reported deliberately under-reporting pain severity to avoid further reduction so that, as they described it, a system intended to make prescribing safer may reduce the accuracy of the clinical information on which safe prescribing depends. Given the association between tapering and subsequent overdose or mental health crisis reported in North American cohorts [21,22,23], under-reporting of this kind, if widespread, would be a patient safety concern that monitoring platforms are not designed to detect. We note that the population-level evidence is genuinely contested, with ecological analysis associating declining prescription rates with declining suicide deaths [24]; our data do not adjudicate that question, but they suggest that explanation and continuity, which are modifiable, were central to how participants described experiencing restriction.

The prominence of nurses in participants’ accounts of continuity deserves comment. Nurses were described as the people who explained decisions, followed up between appointments and noticed absence, yet nurse participants described having little formal authority in this pathway. Given evidence that family and relational support functions as the principal facilitator of adequate analgesia among Arab patients [38], and that culture and language shape pain assessment itself in Saudi settings [39], a formalised nurse-led continuity role is a plausible strategy for addressing erosion of trust without regulatory change. The present data cannot show that such a role would improve disclosure, uptake of alternatives or pain outcomes, and some constraints participants described—distance, supply and cost—lie beyond its reach; it is therefore a proposal requiring development and prospective evaluation.

### 4.4. Conditional, Anticipated Acceptability of Non-Opioid Delivery Systems

Participants’ anticipated appraisals are broadly consistent with discrete choice experiment evidence that patients trade effectiveness against adverse-effect risk, dependence risk, cost and mode of administration [58,59,60], and the qualitative data help to explain the reasoning behind such trade-offs. In participants’ accounts, local action, self-control, discretion and freedom from dependence were valued less as abstract product attributes than as responses to experiences described in Themes 1 to 3. This suggests that the same product may be received differently depending on the relational context of its offer; one clinician participant described a related error, introducing the alternative in the same sentence as the reduction.

Two distinctions are needed in interpreting these appraisals. First, participants’ beliefs about skin-applied formats are perceptions, not pharmacological properties. Topical products such as NSAID gels and the lidocaine 5% plaster act predominantly at or near the site of application with limited systemic exposure [43,45], which is consistent with participants’ reasoning about avoiding the stomach and liver. Transdermal systems, by contrast, are designed to deliver drug into the systemic circulation, and a patch may, itself, contain an opioid, transdermal fentanyl and buprenorphine being established examples [48,49]. A patch is therefore neither intrinsically local nor intrinsically non-opioid, and dependence potential is a property of the agent delivered rather than of the format. The assumption, recurrent in participants’ accounts, that “something on the skin” acts only locally and cannot create dependence was accurate only for some products; this matters for patient education, since it could lead patients to underestimate the systemic effects of transdermal products or to overestimate the reach of topical ones. Second, favourable appraisals do not establish clinical effectiveness, real-world uptake or adherence. Topical NSAIDs are recommended for mild-to-moderate osteoarthritis pain [45], and the lidocaine and high-concentration capsaicin plasters have an established role in localised neuropathic pain [42,43,44], but these indications are narrower than the range of pain participants described, and participants themselves doubted that skin-applied formats could control severe pain. That scepticism reflects participants’ experience and expectations rather than comparative evidence: in the SPACE trial, opioid therapy was not superior to a stepped non-opioid medication strategy for pain-related function over 12 months, pain intensity was slightly better with non-opioid therapy, and medication-related adverse symptoms were more common with opioids [16]. Oral non-opioid options carry their own evidence and cautions [46,47], including the adverse-event burden of gabapentinoids in a setting where they are now controlled [13]; newer delivery technologies [50,51,52,53,54] remain at varying stages of development, with novelty of delivery not guaranteeing superiority [53]; and multimodal and digital adjuncts [55,56,57] would fit the multimodal pathway participants said they were not offered. Whether participants would in fact use these formats, and with what effect, can only be established by studies of actual treatment offers and outcomes.

Two findings here may be novel. The first is the devotional-fit condition. Although compatibility with prayer, ablution and fasting was an interview probe, participants who discussed it described genuine uncertainty about whether an adhesive patch affects the validity of ablution and whether transdermal use raises any issue during fasting, and reported receiving speculation rather than guidance. To our knowledge, this consideration is absent from the analgesic preference literature; it may be relevant to acceptability in Muslim-majority populations and could be addressed by services obtaining and disseminating authoritative guidance, although its influence on actual use is untested. The second is the substitution fear: some participants suspected that alternatives were being introduced in order to justify closing the opioid route (this concern did not correspond to any interview probe and was raised in response to open questions about alternatives). We interpret this suspicion as related to the trust erosion described in Theme 3; if it is more widely shared, it could be a barrier to the non-opioid strategies that balanced-policy frameworks recommend [10], and formulary expansion without attention to trust might not achieve its intended uptake.

### 4.5. Potential Implications and Proposals for Evaluation

At the level of policy, the findings are consistent with arguments for a balanced regulatory approach that addresses both inadequate access to opioids where they are indicated, as in cancer and palliative pain [7,8,9,10], and the uncertain benefit and dose-related harms of long-term opioid therapy for chronic non-cancer pain, as well as with calls for risk-stratified rather than blanket restriction made for gabapentinoids in the same national context [13]. As a purposively sampled qualitative study in one governorate, this study cannot establish that any specific policy change would be effective or feasible. Participants’ accounts nonetheless suggest three proposals that merit evaluation: examining whether the local availability of pharmacies dispensing controlled analgesics in peri-urban and rural areas matches need, now that prescriptions are issued electronically [19,72]; assessing the feasibility and safety of defined, monitored continuation prescribing within primary care for stable patients in whom continued therapy has been judged appropriate, with structured review of benefit and harm; and assessing the cost-effectiveness of formulary coverage for evidence-based non-opioid topical formats, since participants described out-of-pocket alternatives as transferring rather than relieving the burden.

At the level of practice, participants’ accounts suggest four changes that services could consider and evaluate: reviewing dispensing procedures so that controls are not enacted in public view; communicating any dose reduction as an explained clinical decision with a named rationale and an alternative plan, since explanation distinguished a tolerable taper from an experience of abandonment in participants’ accounts, as in previous studies [35,36]; introducing non-opioid delivery systems early and as additions, by a trusted clinician, rather than in the same consultation as a reduction; and obtaining authoritative guidance on the compatibility of topical and transdermal formats with ablution, prayer and fasting. Patient education should also address the difference between topical and transdermal products.

At the level of nursing practice and education, the findings suggest a nurse-led continuity role within the chronic pain pathway that could be developed and evaluated—structured follow-up between appointments, explanation of prescribing decisions, early identification of patients approaching supply exhaustion, and instruction in the correct application of topical and transdermal systems. Curricula in nursing, medicine and pharmacy should address the enactment of stigma at the point of care as an explicit competency, including the credibility asymmetries by gender and age that participants described and that the epistemic injustice literature analyses [32,33,34], and should teach the recognition that culturally patterned under-reporting [38,39] and under-reporting that participants related to the monitoring system can be mistaken for one another.

For research, four priorities follow. First, the substitution fear identified here should be tested at scale, since, if common, it could affect the projected uptake of any non-opioid strategy. Second, discrete choice experiments in Gulf populations should incorporate the attributes participants raised that existing instruments omit—dispensing visibility, devotional compatibility and source of endorsement. Third, a nurse-led continuity intervention of the kind participants described could be specified and should be evaluated prospectively for its effect on trust, disclosure accuracy and pain outcomes. Fourth, the interpretive relationships proposed in Figure 1 should be examined with longitudinal and linked prescribing data, and the anticipated acceptability reported here should be tested against actual uptake when non-opioid formats are offered.

### 4.6. Strengths and Limitations

The study’s principal strengths are its multiperspectival design across seven settings spanning the whole referral pathway, the deliberate inclusion of patients at every point of that pathway, including those who sought but never received opioid analgesia, analysis conducted in the participants’ own language with independent back-translation of published quotations, and the separation of recruitment from clinical care to reduce the risk that participants would fear consequences for their prescriptions.

Several limitations qualify the findings. The study was conducted in a single governorate, and although Al-Ahsa’s mix of urban and rural settlement is representative of much of the Eastern Province, patterns of access elsewhere in the Kingdom—particularly in Riyadh and Jeddah, where specialist density is higher—may differ. Participation was voluntary, and it is plausible that those with the strongest experiences of restriction, in either direction, were most motivated to take part; patients who disengaged from services altogether are by definition absent, and their accounts may differ from those reported here. Participants’ accounts spanned the period before and after the national transition to electronic prescribing and dispensing in 2025, and facility-level implementation was not documented, so the findings cannot be attributed to a specific version of the regulatory system. Eligible patients were identified by staff of the services under study, which may have introduced gatekeeping, since patients with strained relationships with those services may have been less likely to be approached; although staff logged the patients they considered (Section 2.5), the judgement they exercised in compiling clinic lists could not be audited. Interviews were conducted in several modalities, which may have influenced disclosure. Because the interview guide probed many of the topics that became sub-themes, the findings reflect both participants’ own framings and the questions they were asked. All data are self-reported and were not triangulated against prescription records, which would have permitted verification of tapering histories but would also have compromised the separation between research and clinical care that the design prioritised. Patients with a treated substance use disorder were excluded to keep the analytic focus clear, but that group experiences restriction acutely and their exclusion is a real boundary on transferability. Transcripts were not returned to participants, for reasons given in Section 2.8, and participants did not review the developed themes; checking was limited to in-interview summaries and clarification calls, which support clarification of meaning but are not equivalent to participant feedback on the findings [69,73]. Appraisals of non-opioid delivery systems were elicited from participants who had, in most cases, not used the formats discussed, partly in response to a visual card; they represent anticipated acceptability and conditions for hypothetical use, and the study included no choice task, treatment offer or follow-up of uptake. Finally, in accordance with reflexive thematic analysis the themes are constructions of this research team, whose professional positions are described in Section 2.10; a differently composed team might have foregrounded different patterns.

## 5. Conclusions

Adults living with chronic non-cancer pain in Al-Ahsa described opioid restriction less as a matter of dose than of relationship: an access pathway that made relief contingent on referral, authorisation and distance; encounters in which they felt recategorised as suspects; and tapers or refusals that, when unexplained, were experienced as abandonment. In the research team’s interpretation, participants’ adaptations to an unreliable supply risked being read as signs of risk, and institutional distrust left interpersonal trust in named clinicians—often nurses—as the main basis of their engagement with the pathway.

Against this background, participants expressed conditional, anticipated openness to non-opioid drug-delivery systems, valuing perceived local action, self-control, discretion and freedom from the moral weight of dependence. They set conditions that a formulary decision alone would not satisfy: affordability within governmental coverage, dependable local supply, honest acknowledgement of the limits of skin-applied formats in severe pain, clear guidance on compatibility with ablution, prayer and fasting, and endorsement offered early by a trusted clinician rather than at the moment of refusal. Participants’ fear that alternatives might be offered to justify closing the opioid route suggests that these conditions concern trust as much as pharmacology. These are stated, hypothetical appraisals and do not establish how patients would respond to an actual offer.

Because the long-term benefit of opioid therapy for chronic non-cancer pain is uncertain and its harms are dose-related [14,15,16], these findings do not argue for wider opioid prescribing; they concern how restriction is organised, explained and accompanied by credible alternatives. Restrictive policy in low-consumption settings may therefore warrant evaluation not only by the prescribing trends associated with it but by whether patients within it feel treated as credible. The proposals arising from this study—risk-stratified rather than blanket restriction, dispensing processes that do not publicly mark patients, explanation and an alternative plan whenever a dose is reduced, attention to formulary coverage of evidence-based non-opioid formats, and a defined nurse-led continuity role—are consistent with the patient-centred commitments of the Health Sector Transformation Program [17], but they require development and evaluation before their feasibility or effectiveness can be assumed.

## Figures and Tables

**Table 1:** Characteristics of participants (patients, n = 30; clinicians, n = 12).

Characteristic	Category	n	%
PATIENTS (n = 30)			
Sex	Male	16	53.3
	Female	14	46.7
Age (years)	18–34	5	16.7
	35–49	9	30.0
	50–64	11	36.7
	≥65	5	16.7
Primary pain condition	Chronic low back/musculoskeletal pain	13	43.3
	Peripheral neuropathic pain	8	26.7
	Chronic post-surgical pain	5	16.7
	Mixed/widespread pain	4	13.3
Duration of chronic pain	6 months–2 years	6	20.0
	>2–10 years	17	56.7
	>10 years	7	23.3
Current analgesic status	Currently prescribed an opioid	11	36.7
	Tapered or discontinued in past 24 months	10	33.3
	Sought but never prescribed an opioid	9	30.0
Residence	Urban (Hofuf/Mubarraz)	19	63.3
	Peri-urban or rural village	11	36.7
Nationality	Saudi	23	76.7
	Non-Saudi resident	7	23.3
Payment route	Governmental (free at point of use)	18	60.0
	Private out-of-pocket	7	23.3
	Employer or private insurance	5	16.7
Employment status	In paid employment	12	40.0
	Not working due to pain	9	30.0
	Retired or homemaker	9	30.0
CLINICIANS (n = 12)			
Professional group	Pain medicine/anaesthesia physician	3	25.0
	Family/primary care physician	3	25.0
	Registered nurse	3	25.0
	Clinical or dispensing pharmacist	3	25.0
Setting	Tertiary or university hospital	6	50.0
	Primary health care centre	3	25.0
	Private hospital or authorised pharmacy	3	25.0
Experience in current role	1–5 years	4	33.3
	>5–15 years	5	41.7
	>15 years	3	25.0

Percentages are calculated within each participant group and may not total 100 due to rounding.

**Table 2:** Themes, sub-themes and analytic descriptions.

Theme	Sub-Theme	Description
1. “A door with many locks”: navigating a restricted access pathway	1.1 Bureaucratic gatekeeping and the geography of the controlled prescription	Specialist-only prescribing; non-transferable controlled prescriptions; and, in participants’ experience, few dispensing points were described as turning analgesia into an administrative and logistical project undertaken in pain.
	1.2 Rationing, running out and the arithmetic of the last tablet	Fixed quantities, absent refills and appointment delays were linked by participants to splitting doses, stockpiling and planning work, travel and family life around refill dates.
2. “As if I came to steal”: stigma at the point of care	2.1 Being read as drug-seeking in clinics, emergency rooms and pharmacies	Patients describe suspicion enacted through questioning, delay, documentation checks and non-verbal signals, most acutely in emergency and dispensing settings.
	2.2 Anticipated stigma, concealment and proving legitimate pain	Anticipated suspicion was described alongside concealment from family, employers and clinicians, and alongside rehearsed strategies for demonstrating that pain and need are genuine; some women and younger men reported credibility gaps.
3. “I trust the person, not the system”: erosion and repair of therapeutic trust	3.1 Unexplained tapering and the experience of being left mid-treatment	Dose reduction communicated as an external rule rather than a clinical decision was experienced as abandonment and was described alongside concealment, self-medication and disengagement.
	3.2 Continuity, explanation and the clinicians who restore trust	Named, continuous clinicians who explain constraints and share decisions were described as repairing trust locally, even where confidence in the wider system remains absent; clinicians’ own fear of regulatory sanction is visible to patients.
4. “Something I can put on my skin”: anticipated acceptability and conditions for hypothetical use of non-opioid delivery	4.1 Valued attributes: local action, self-control, discretion and freedom from dependence	Topical, transdermal and localised delivery formats were valued for being perceived to act where the pain is, being controllable by the patient, attracting no suspicion and being perceived to carry no dependence risk.
	4.2 Conditions for anticipated use: cost, supply, credibility and clinician endorsement	Stated, hypothetical willingness to use these formats was conditional on affordability, dependable local availability, believable efficacy for severe pain, compatibility with prayer, ablution and fasting practices, and explicit endorsement by a trusted clinician.

Most sub-theme topics were explored directly through interview probes (Supplementary Material S1); the themes, their organising concepts and the relationships between them were constructed analytically by the research team.

**Table 3:** Attributes of non-opioid drug delivery systems as valued, questioned and rejected by patient participants (anticipated appraisals).

Attribute Domain	What Participants Valued	What Participants Questioned or Rejected
Site of action	Delivery “to the place itself”; perceived avoidance of stomach, liver and whole-body exposure	Doubt that a locally applied agent could reach deep, radiating or widespread pain
Control and autonomy	Self-application and self-removal; a decision the patient can make without permission	Concern about correct application, skin reactions and what to do if a patch is dislodged
Visibility and discretion	No controlled register, signature or supervisor at the point of purchase; no public marking as a controlled-drug user	Visibility of a patch on exposed skin during work, prayer or family gatherings
Dependence and moral weight	Absence of dependence connotation; relief from family and religious concern about attachment to a medicine	Suspicion that “if it is really strong, it must also be dangerous”, transferring opioid concerns to new formats
Perceived potency	Acceptable and welcome for mild to moderate or localised pain, and as an adjunct	Scepticism that any topical or transdermal format can control severe or crisis pain; fear it will be offered as a replacement rather than an addition
Cost and coverage	Willingness to pay something for autonomy and discretion	Out-of-pocket cost of repeat applications; uncertainty about insurance and governmental formulary coverage; perceived unaffordability over years of use
Availability and supply	Preference for formats obtainable from a local pharmacy without travel	Fear of intermittent stock, discontinued lines and having to return to the restricted pathway when supply fails
Cultural and religious fit	Compatibility with daily routine when discreetly sited; acceptable to family	Uncertainty about ablution and prayer with an adhesive patch, and about use during fasting; desire for explicit guidance
Source of endorsement	Explicit recommendation by a known, trusted clinician; demonstration of correct use	Distrust of pharmacy or media promotion; association of unendorsed alternatives with traditional or unproven remedies

Attributes were constructed from patient accounts, including responses to interview probes (Supplementary Material S1), and are anticipated appraisals of formats most participants had not used; the table is an analytic summary and not a frequency count.

## Data Availability

The datasets generated and analysed during this study are not publicly available because they consist of individual qualitative interview transcripts containing contextual detail that could permit identification of participants within a defined regional health system and because participants did not consent to public deposition of full transcripts. De-identified excerpts additional to those presented here are available from the corresponding author upon reasonable request, subject to approval by the Research Ethics Committee of King Faisal University.

## Supplementary Materials

The following supporting information can be downloaded at: https://www.mdpi.com/article/10.3390/healthcare14203379/s1. The following supporting information is available as separate files: Supplementary Material S1: Semi-Structured Interview Guide (patient and clinician versions), reproduced as used during data collection and therefore retaining the study’s original working title (see Section 2.7); Supplementary Material S2: Completed COREQ (32-item) checklist, presented under the revised article title and aligned with the methods reported in Section 2.1, Section 2.2, Section 2.3, Section 2.4, Section 2.5, Section 2.6, Section 2.7, Section 2.8, Section 2.9, Section 2.10 and Section 2.11; Supplementary Material S3: Visual stimulus card shown to patient participants (interview guide, Question 18).

## Abbreviations

COREQ: Consolidated Criteria for Reporting Qualitative Research; NSAID: non-steroidal anti-inflammatory drug; RTA: reflexive thematic analysis; SFDA: Saudi Food and Drug Authority.
