# Supplementary material for: Patient Experiences of Access, Stigma, and Trust and Preferences for Non-Opioid Drug Delivery Systems in Chronic Pain Management
Source: Healthcare (Basel). 2026 Oct 10;14(20):3379. doi: 10.3390/healthcare14203379 (PMC13704753; doi:10.3390/healthcare14203379)
Supplement: Supplementary file 1 [file healthcare-14-03379-s001.zip › healthcare-4577485-supplementary S1-triage.pdf]

**Supplementary Material S1**

**Semi-Structured Interview Guide (Patient and Clinician Versions)**

**Study title:** When Opioids Are Restricted: Patient Experiences of Access, Stigma, and Trust and Preferences for Non-Opioid Drug Delivery Systems in Chronic Pain Management—A Qualitative Study from the Al-Ahsa Region, Saudi Arabia

**Design:** Multiperspectival qualitative study; individual semi-structured interviews; reflexive thematic analysis

**Setting:** Seven public and private health settings, Al-Ahsa Governorate, Eastern Province, Saudi Arabia

**Participants:** 30 adults with chronic non-cancer pain (P01–P30); 12 clinicians (C01–C12)

**Period:** 4 May – 26 June 2026

**Languages:** Gulf colloquial Arabic, Modern Standard Arabic, English

**Guide version:** Version 2.1, finalised after piloting with three participants

## 1. General Instructions for the Interviewer

1. Conduct the interview in the language the participant chooses. Do not correct or standardise the participant's dialect; record idiom as spoken, since idiomatic phrasing is analytically important in this study.
2. State clearly at the outset that you have no role in the participant's clinical care, that nothing said in the interview will be entered in their file or shared with their treating team, and that participation, refusal or withdrawal has no effect whatsoever on their treatment or prescriptions.
3. Use the domains below as a flexible map, not a script. Follow the participant's account where it leads; return to unasked domains later. It is acceptable to change the order entirely.
4. Ask open questions first and probes only afterwards. Never introduce the words "addiction", "dependence", "abuse" or "drug-seeking" before the participant does; if the participant uses such a term, ask what they mean by it in their own words.
5. Do not offer clinical advice, opinions on the participant's regimen, or reassurance about regulation. If asked a clinical question, note it and offer a referral back to the treating service after the interview.
6. Allow silence. Participants describing suspicion or refusal frequently pause before the most significant material; do not fill the pause with a further question.
7. Domain 5 (alternatives) uses a neutral visual card showing generic delivery formats. Ask all open questions in Domain 5 before showing the card, so that the participant's spontaneous frame of reference is captured first.
8. Apply the distress protocol if the participant becomes upset: pause, offer a break, offer to stop or to continue off-record, and—with the participant's agreement—offer referral to the treating service. Record the activation in the field notes.
9. Write field notes within two hours of the interview, covering setting, interruptions, non-verbal communication, anything said before or after the recording, and your own immediate reactions.

## 2. Consent and Opening Checklist

- ☐ Participant has read, or had read to them, the participant information sheet, and has had at least 48 hours to consider participation.
- ☐ Purpose, voluntariness and the right to withdraw at any point without giving a reason have been explained verbally.

☐ It has been stated explicitly that participation has no bearing on current or future treatment, prescriptions or employment.

☐ Confidentiality, pseudonymisation and secure separate storage of audio and transcripts have been explained.

☐ Specific consent for audio-recording obtained and recorded on the consent form.

☐ Specific consent for publication of anonymised verbatim quotations obtained.

☐ For participants unable to read: information sheet read aloud in full in the presence of an impartial witness who has countersigned.

☐ Written consent form signed and dated; a copy given to the participant.

☐ Participant has been offered the opportunity to ask questions, and these have been answered.

☐ Recording started; date, time, setting, participant code and language of interview stated on the recording.

Opening question (used to settle the participant before the domains begin): “Before we talk about medicines and hospitals at all — can you tell me about your pain? When it started, and what an ordinary day with it is like for you now.”

## 3. Interview Questions

Twenty core questions across five domains. Probes are prompts of last resort, to be used only where the participant's account does not already cover the ground.

**Table S1.** Core interview questions and probes, by domain (patient version).

| Domain | Q | Interview question | Probes / interviewer notes |
|---|---|---|---|
| **1. The pain story and its impact** | **1** | How would you describe your pain to someone who has never had it? | *Location, character, fluctuation over a day and a week. Do not offer a numeric scale; if the participant uses one, ask what that number means to them.* |
| | **2** | What has this pain changed about your life — your work, your family, your worship, the things you used to do? | *Work and income; household roles; attendance at family and religious gatherings; sleep; what they have given up and what they have kept.* |
| | **3** | Who knows about your pain, and who does not? | *Family, employer, friends, neighbours. Reasons for telling or not telling. Explore without implying that concealment is expected.* |
| | **4** | Looking back, what have you tried for it over the years? | *Prescribed, over-the-counter, physiotherapy, injections, surgery, traditional or herbal remedies, religious practices. Ask what helped and what was abandoned, and why.* |
| **2. The pathway to obtaining pain medication** | **5** | Walk me through what actually has to happen for you to get your pain medicine — from the beginning. | *Let the participant narrate the whole sequence uninterrupted first. Then map: who refers, who prescribes, where it is dispensed, how long each step takes.* |
| | **6** | How far do you have to travel, and what does the journey cost you — in time, money, and pain? | *Distance to prescriber and to authorised dispensing site; transport; who accompanies them; work time lost; effect of the journey on the pain itself.* |
| | **7** | What happens when the medicine runs out before the next prescription is due? | *Frequency; what they do; who they turn to; whether they adjust doses in advance. Do not evaluate any strategy described.* |
| | **8** | Has the amount or type of your pain medicine ever been changed or stopped? Tell me about that. | *Who told them; what reason was given; whether an alternative was offered; whether they were consulted; what happened afterwards.* |
| **3. Experiences at the point of prescribing and dispensing** | **9** | Tell me about a time in a clinic or hospital when you felt you were treated well about your pain. | *Ask for a specific occasion rather than a general impression. What exactly did the person do or say?* |
| | **10** | And a time when you felt you were not treated well? | *Setting (clinic, emergency, ward); what was said and not said; body language; who else was present; what the participant did afterwards.* |
| | **11** | What is it like for you at the pharmacy when you collect this medicine? | *Waiting; the controlled register; countersignatures; supervisors; other customers in the queue; whether they have changed which pharmacy they use.* |

| Domain | Q | Interview question | Probes / interviewer notes |
|---|---|---|---|
| | **12** | Do you feel you have to prove anything when you ask for help with your pain? If so, how do you do that? | *Documents carried; how they dress or speak; whom they bring; what they avoid saying. Explore differences by sex, age and nationality if the participant raises them.* |
| **4. Trust and the therapeutic relationship** | **13** | Who, if anyone, do you trust about your pain — and what did they do to earn that? | *Named individuals versus institutions; role (physician, nurse, pharmacist); continuity over time; what specifically was said or done.* |
| | **14** | Is there anything about your pain or your medicines that you do not tell the health professionals? What makes that difficult? | *Severity; extra doses; medicines obtained elsewhere; use of family members' medicines. Approach without judgement; reiterate confidentiality first.* |
| | **15** | When decisions are made about your pain medicine, how are they explained to you? | *Whether the reason is clinical or presented as an external rule or instruction; whether alternatives were discussed; whether they felt part of the decision.* |
| | **16** | What would need to change for you to feel confident in the care you get for your pain? | *Distinguish what would need to change in individual behaviour from what would need to change in the system. Ask what is realistic.* |
| **5. Non-opioid and alternative delivery systems** | **17** | Apart from tablets, what other ways of giving pain medicine have you heard of or used? | *Ask before showing the visual card. Record the spontaneous frame of reference, including traditional and non-pharmacological options.* |
| | **18** | [Show card.] Looking at these different forms — a plaster or patch, a gel or cream, an injection, an implanted device — what appeals to you, and what does not? | *Take each format in turn. Probe reasons rather than ranking. Note where preference is about the medicine and where it is about the encounter.* |
| | **19** | If your doctor offered you one of these instead of, or alongside, your current medicine, what would you want to know first? | *Efficacy for their level of pain; side effects; cost; availability; how to apply and remove; what happens if it fails; whether it replaces or adds to current treatment.* |
| | **20** | What would have to be true for you to actually use one of these — and what would stop you? | *Cost and coverage; local availability and continuity of supply; who recommends it; visibility to others; compatibility with prayer, ablution and fasting; family acceptance.* |

*Card shown at Q18 depicts unbranded, generic representations of delivery formats only; no product names, manufacturers or claims appear on it.*

## 4. Clinician Version — Additional and Substituted Questions

Clinician interviews used Domains 2 to 5 with the wording adapted to professional perspective and replaced Domain 1 with the questions below. Clinicians were asked to speak about the pathway and about patterns they observed, not about identifiable patients.

**Table S2.** Domain 1 replacement and additional questions (clinician version).

| Q | Interview question | Probes / interviewer notes |
|---|---|---|
| **C1** | Describe your role in the care of patients with chronic non-cancer pain, and how long you have worked in it. | *Setting; caseload; where they sit in the referral or dispensing pathway; what they can and cannot authorise.* |
| **C2** | Walk me through what happens, from your side, when a patient with chronic non-cancer pain needs a controlled analgesic. | *Referral, authorisation, documentation, monitoring platform, dispensing. Where the process most often stalls, and why.* |
| **C3** | What constraints do you work within when prescribing or dispensing these medicines, and how do they affect your decisions? | *Regulation, institutional policy, audit, professional risk. Ask what they would do differently if the constraint were removed.* |
| **C4** | How do you decide whether a request for pain medication is clinically appropriate? | *Explicit criteria versus impression. Invite reflection on what informal cues are used, and on how these might affect different patients.* |
| **C5** | How do you approach the conversation when you have to reduce, refuse or stop a patient's opioid analgesia? | *Wording used; reason given; whether an alternative is offered at the same time; how patients typically respond; what they have learned to do differently.* |
| **C6** | In your experience, which patients find this pathway hardest, and why? | *Rural residence, sex, age, nationality, payment route, literacy, absence of a family advocate.* |
| **C7** | What non-opioid options are actually available to you here, and what determines whether you offer them? | *Formulary and coverage; supply and stock continuity; cost to the patient; own confidence in the evidence; time available to explain and demonstrate.* |
| **C8** | What would need to change — in regulation, in the service, or in your own practice — for chronic pain to be managed better in this region? | *Ask for concrete, implementable changes and for their view on which are realistic within current policy.* |

## 5. Closing the Interview

1. Summarise back to the participant, in two or three sentences, the main things you have understood from them, and ask whether you have understood correctly. Record any correction.
2. Ask: “Is there anything important about this that I did not ask you about?” Allow time; this question frequently produces significant material.
3. Ask whether there is anything said during the interview that the participant would prefer not to be used, and record any such request.
4. Confirm that quotations will be published without any name, facility or identifying detail, and remind the participant of the withdrawal window.
5. Ask permission to make a brief follow-up telephone call if a point needs clarification, and record the answer.
6. Thank the participant. Stop the recording, and state on the recording that it has ended.
7. Complete the demographic form below with the participant before they leave, so that the interview itself is not opened with demographic questions.

8. Write field notes within two hours.

**6. Demographic Data Collection (Completed After the Interview)**

Collected after the interview to avoid opening the encounter in an administrative register. All fields are optional, and the participant may decline any item.

**Table S3.** Post-interview demographic and clinical record form.

| Item | Response |
|---|---|
| Participant code (P01–P30 / C01–C12) | |
| Date, start and end time of interview | |
| Setting of interview (facility private room / telephone / participant's home / video call) | |
| Language of interview | |
| Age (completed years) | |
| Sex | |
| Nationality (Saudi / non-Saudi resident) | |
| Marital status and household composition | |
| Highest level of education completed | |
| Employment status (in paid work / not working due to pain / retired / homemaker / student) | |
| Residence (urban Hofuf or Mubarraz / peri-urban / rural village) and approximate distance to prescribing facility | |
| Primary pain condition and site | |
| Duration of chronic pain (months or years) | |
| Current analgesic regimen (all prescribed and non-prescribed analgesics) | |
| Current analgesic status (currently prescribed an opioid / tapered or discontinued in past 24 months / sought but never prescribed) | |
| Any tapering or discontinuation in the past 24 months, and approximate date | |
| Payment route (governmental / private out-of-pocket / employer or private insurance) | |
| Usual prescribing facility and usual dispensing pharmacy | |

| Item | Response |
| --- | --- |
| Previous or current use of any topical or transdermal analgesic (specify) | |
| Field-note reference number | |
| CLINICIAN ONLY: professional group, setting, years in current role, prescribing or dispensing authority for controlled analgesics | |
