# Supplementary material for: Patient Experiences of Access, Stigma, and Trust and Preferences for Non-Opioid Drug Delivery Systems in Chronic Pain Management
Source: Healthcare (Basel). 2026 Oct 10;14(20):3379. doi: 10.3390/healthcare14203379 (PMC13704753; doi:10.3390/healthcare14203379)
Supplement: Supplementary file 1 [file healthcare-14-03379-s001.zip › healthcare-4577485-supplementary S2-triage.pdf]

Supplementary Material S2

Patient Experiences of Access, Stigma, and Trust and the Anticipated Acceptability of Non-Opioid Drug Delivery Systems in Chronic Pain Management

Completed COREQ checklist (Consolidated Criteria for Reporting Qualitative Research; Tong, Sainsbury and Craig, 2007) — revised version aligned with the methods reported in the revised manuscript. Revised entries are highlighted in yellow.

No.	Item	Guide question/description	Reported in this study	Location in manuscript
**Domain 1: Research team and reflexivity — Personal characteristics**				
1	Interviewer/facilitator	Which author(s) conducted the interview or focus group?	Patient interviews were conducted by M.S.; clinician interviews were conducted by S.I.A.	Section 2.6
2	Credentials	What were the researcher's credentials?	M.S.: PhD-prepared nursing academic/researcher. S.I.A.: physician-researcher.	Sections 2.6, 2.10
3	Occupation	What was their occupation at the time of the study?	M.S.: nursing academic/researcher (Gerontological Nursing Department, Faculty of Nursing, Cairo University). S.I.A.: physician-researcher, College of Medicine, King Faisal University, Al-Ahsa.	Title page; Section 2.10
4	Gender	Was the researcher male or female?	Both interviewers were male.	Section 2.10
5	Experience and training	What experience or training did the researcher have?	M.S. has a longstanding programme of qualitative research on pain and ageing in Arab populations, including prior qualitative work on barriers to pain management in elderly Arab patients. S.I.A. is a physician-researcher based in Al-Ahsa with familiarity with the regional service configuration.	Section 2.10
**Domain 1 — Relationship with participants**				
6	Relationship established	Was a relationship established prior to study commencement?	No. No participant had a prior clinical or supervisory relationship with the interviewers, and neither interviewer had clinical or supervisory responsibility for the participants they interviewed.	Sections 2.5, 2.6
7	Participant knowledge of the interviewer	What did the participants know about the researcher?	Participants received the information sheet and a full explanation of the study, including that the research was separate from their clinical care and would have no bearing on treatment, prescriptions or employment.	Sections 2.5, 2.11
8	Interviewer characteristics	What characteristics were reported about the interviewer/facilitator?	Professional backgrounds, potential insider assumptions and the risk of "hearing endurance where participants were describing constraint" are reported, with the use of a reflexive journal and analytic memos to interrogate system-centred readings.	Section 2.10
**Domain 2: Study design — Theoretical framework**				
9	Methodological orientation and theory	What methodological orientation was stated to underpin the study?	Reflexive thematic analysis (Braun and Clarke) conducted at a latent level with a combined orientation. Coding remained open to meanings not anticipated by the interview guide, but data generation was deliberately structured by theoretically informed probes	Sections 2.1, 2.7, 2.9

No.	Item	Guide question/description	Reported in this study	Location in manuscript
			(Supplementary Material S1), and the analysis was sensitised to the concepts of stigma and epistemic injustice. The topics that make up most sub-themes were therefore explored directly through the interview guide; the analysis generated the organisation of these topics into themes, the meanings participants attached to them and the interpretive links between them. The analysis is not described as primarily inductive.	
**Domain 2 — Participant selection**				
10	Sampling	How were participants selected?	Purposive maximum-variation sampling for both samples. Patients: sex, age band, pain condition, pain duration, analgesic status (currently prescribed an opioid; tapered/discontinued; sought but never prescribed), urban/rural residence, nationality and payment route. Clinicians: professional group and setting.	Section 2.3; Table 1
11	Method of approach	How were participants approached?	Patients were identified by clinical staff not involved in the study, who provided the information sheet and asked permission for contact; interested patients were telephoned by the research team and given at least 48 hours to decide. Clinicians were invited directly through service leads.	Section 2.5
12	Sample size	How many participants were in the study?	42 participants: 30 patients (P01–P30) and 12 clinicians (C01–C12).	Section 2.3; Table 1
13	Non-participation	How many people refused to participate or dropped out? Reasons?	Four patients who were contacted declined (time constraints, n = 3; unwillingness to discuss medication use, n = 1). Screening log: 47 potentially eligible patients listed; 13 not approached (variation category already filled, n = 8; could not be reached, n = 3; judged acutely unwell by the treating team, n = 2); 34 contacted; 30 participated. All 12 invited clinicians agreed to participate. No participant withdrew after consent.	Sections 2.3, 2.5
**Domain 2 — Setting**				
14	Setting of data collection	Where was the data collected?	Patients chose the location: private room within the participating facility (n = 19), telephone (n = 7) or participant's home (n = 4). Clinician interviews took place in private offices or by video call. Seven settings in Al-Ahsa Governorate.	Sections 2.2, 2.6
15	Presence of non-participants	Was anyone else present besides the participants and researchers?	No non-participants were present during interviews. For the two participants unable to read, an impartial witness was present during the consent process only.	Section 2.11
16	Description of sample	What are the important characteristics of the sample?	Demographic, clinical and service characteristics are given in Table 1; opioid agents, treatment duration, pattern of use and what never-prescribed participants had sought are reported in Section 3.1.	Section 3.1; Table 1
**Domain 2 — Data collection**				
17	Interview guide	Were questions, prompts, guides provided by the authors? Was it pilot tested?	Yes. Semi-structured guide developed from the literature and reviewed by two bilingual clinicians and one patient adviser; theoretically informed probes are listed in Section 2.7. Piloted with three patients; two questions were reworded and the pilot interviews were	Section 2.7; Supplementary Materials S1 and S3

No.	Item	Guide question/description	Reported in this study	Location in manuscript
			retained. A neutral visual card was used at Question 18. The guide is reproduced as used in the field and retains the study's original working title.	
18	Repeat interviews	Were repeat interviews carried out? If yes, how many?	No repeat interviews. Eight participants were re-contacted by telephone for clarification of specific statements.	Section 2.6
19	Audio/visual recording	Did the research use audio or visual recording to collect the data?	Audio-recorded with written consent.	Sections 2.8, 2.11
20	Field notes	Were field notes made during and/or after the interview?	Yes; written within two hours of each interview (setting, non-verbal communication, interruptions, interviewer's immediate impressions) and used analytically; interview modality was recorded for every transcript.	Section 2.6
21	Duration	What was the duration of the interviews?	Patients: 42–88 minutes (mean 61). Clinicians: 35–55 minutes (mean 44).	Section 2.6
22	Data saturation	Was data saturation discussed?	Data saturation was not used as a stopping rule, consistent with reflexive thematic analysis. Sample adequacy was judged using information power at analytic meetings during data generation.	Section 2.3
23	Transcripts returned	Were transcripts returned to participants for comment and/or correction?	No. Transcripts were not returned to participants, because pilot feedback indicated that this would be burdensome and might inhibit candour.	Section 2.8
**Domain 3: Analysis and findings — Data analysis**				
24	Number of data coders	How many data coders coded the data?	Two researchers coded the first eight transcripts independently and then coded collaboratively; divergent readings were recorded in analytic memos and discussed.	Section 2.9
25	Description of the coding tree	Did authors provide a description of the coding tree?	Yes: 412 initial codes (patient corpus) and 96 (clinician corpus), clustered by visual mapping; two candidate themes (financial burden; family involvement) were dissolved and redistributed. Final themes and sub-themes are summarised in Table 2; coding framework versions are held in the audit trail.	Sections 2.9, 2.10; Table 2
26	Derivation of themes	Were themes identified in advance or derived from the data?	Themes were constructed analytically through the six phases of reflexive thematic analysis; they were not specified in advance. However, most sub-theme topics were explored directly through theoretically informed interview probes (Supplementary Material S1), so findings on these topics reflect deliberate exploration rather than spontaneous emergence. What the analysis generated was the organisation of these topics into themes, the meanings participants attached to them and the interpretive links between them (presented as the research team's interpretive model in Figure 1). Themes were developed from the patient corpus; clinician accounts were mapped onto them and points of divergence are reported.	Sections 2.7, 2.9, 3.2, 3.7; Table 2; Figure 1
27	Software	What software, if applicable, was used to manage the data?	NVivo 14.	Section 2.9

No.	Item	Guide question/description	Reported in this study	Location in manuscript
28	Participant checking	Did participants provide feedback on the findings?	No. Participants did not provide feedback on the developed themes or the final analytic findings. Immediate understanding was checked through end-of-interview summaries, in which the interviewer summarised key points for the participant to confirm or correct, and through eight clarification calls about specific statements. These procedures supported clarification of meaning during or shortly after interviews and are not equivalent to participant feedback on the findings.	Sections 2.8, 2.10, 4.6
**Domain 3 — Reporting**				
29	Quotations presented	Were participant quotations presented to illustrate the themes? Was each quotation identified?	Yes. Patient quotations are identified by participant code, sex and age band; clinician quotations by code and professional group. Descriptors were reduced after an assessment of deductive-disclosure risk.	Sections 2.11, 3.3–3.6
30	Data and findings consistent	Was there consistency between the data presented and the findings?	Yes. Findings are grounded in extracts; frequency terms are descriptive rather than counts; convergence and divergence between patient and clinician accounts are reported.	Sections 2.9, 3.3–3.7
31	Clarity of major themes	Were major themes clearly presented in the findings?	Four themes, each with two sub-themes, are presented in Table 2 and Sections 3.3–3.6.	Table 2; Sections 3.3–3.6
32	Clarity of minor themes	Is there a description of diverse cases or discussion of minor themes?	Yes, including participants who consumed supply early rather than rationing (sub-theme 1.2), variation in how patients interpreted clinicians' constraint (sub-theme 3.2), and clinician accounts that diverged from patient accounts (Section 3.7).	Sections 3.3.2, 3.5.2, 3.7
