# Supplementary material for: Patient Experiences of Access, Stigma, and Trust and Preferences for Non-Opioid Drug Delivery Systems in Chronic Pain Management
Source: Healthcare (Basel). 2026 Oct 10;14(20):3379. doi: 10.3390/healthcare14203379 (PMC13704753; doi:10.3390/healthcare14203379)
Supplement: Supplementary file 1 [file healthcare-14-03379-s001.zip › healthcare-4577485-supplementary S3-triage.pdf]

Supplementary Material S3

Visual stimulus card shown to patient participants (patient interview guide, Question 18)

Different ways that medicines for pain can be given

طرق مختلفة لإعطاء أدوية الألم

A	B	C	D	E
Tablet or capsule taken by mouth<br>قرص أو كبسولة تؤخذ عن طريق الفم	Medicated plaster or patch applied to the skin<br>لصقة طبية توضع على الجلد	Gel or cream rubbed onto the skin<br>جل أو كريم يُدهن على الجلد	Injection<br>حقنة	Small device placed under the skin or near the spine<br>جهاز صغير يوضع تحت الجلد أو بالقرب من العمود الفقري

The card was offered only after participants had answered open-ended questions about alternatives. It names delivery formats only: it does not name any medicine; state whether a format contains an opioid; or describe effectiveness, side effects, cost or availability. Formats were presented in a fixed order with identical layout and no images or colours that might suggest a preferred option. Interviewers asked, “Have you used or heard of any of these?” and “What would you think if one of these were suggested for your pain?”
